# The Pilgrimage of Thought
*ψϒXH: a Discourse on the Birth and Pilgrimage of Thought. By Walter Cooper Dendy, Honorary Fellow and formerly President of the Medical Society of London, Corresponding Fellow of the Academy of Surgery at Madrid, &c. London: Longman. 1853.


**Published:** 1854-01-01

**Authors:** 


### Art. VI.—THE PILGRIMAGE OF THOUGHT.*

The possession of knowledge does not necessarily imply the power of communicating it. It is notorious that many of the most eminent philosophers have been unable to express in simple and familiar language the recondite truths which they may have mastered. Hence the harsh and uncouth technicalities and ponderous sentences which abound in professedly learned works. Others, again, enjoy an extraordinary facility of expression, which we have often thought must depend on some special faculty, for it is not acquired by education, nor can all the *limæ labor* in the world produce it. This fortunate gift the author before us, Mr. Dendy, enjoys in a high degree; upon the most abstruse subjects he writes perspicuously, fluently, and gracefully, and in the obscure regions of metaphysics hê has the happy knack, like the blind old man in Werter, even in the depth of winter, of discovering flowers. He sets out in the full enjoyment of the cheerful faith that—

> "There's a divinity that shapes our ends,
> Rough-hew them how we will;"—

and he communes with our spiritual nature as though he were privileged to sit in the Cave of Plato, familiarly taxing the mysteries of his own soul. In this inquisitive, and by no means unphilosophical mood, he proceeds to analyze the mental alchemy of our being, determining, if possible, to arrive at first principles. "What," he asks, "is the fountain of that thought—what that mysterious light that has so richly illumined the framework of human nature — that inspired Shakspeare to create new worlds of fancy—Milton to presume an earthly guest in Paradise and Pandemonium—Herschel to bring down to us the stars of heaven—Priestley and Davy and Wollaston and Faraday, to play their splendid tricks with gases and metals—Smeaton, Telford, and Stephenson to subjugate the stubborn laws of mechanics to their will, and Newton to demonstrate the ruling principle of the universe?" (p. 1.)

* ΨΥΧΗ: a Discourse on the Birth and Pilgrimage of Thought. By Walter Cooper Dendy, Honorary Fellow and formerly President of the Medical Society of London, Corresponding Fellow of the Academy of Surgery at Madrid, &c. London: Longman. 1853.

Whence, indeed, we may truly ask, is this mystic and far-searching principle derived? Who can trace, as the noble poet finely expresses it, "home to its cloud this lightning of the mind?" What is the origin and nature of the thought which puzzles us? How is it connected with the material substance of the brain or nervous system? What are the laws that govern its birth and pilgrimage? Is it material or immaterial?—and what am I who thus catechize the ideas and perceptions of which I am conscious? What, as Descartes asked more than two hundred years ago, is this *ego?* Am I anything or am I nothing? am I anybody or nobody? What answer does that profound reasoner, Pascal, give to these curious cross-questionings? "Man," says he, "is to himself the most astonishing object in nature, for he cannot conceive what body is, still less what spirit is, and less than all how a body and a spirit can be united. This is the climax of his difficulties, yet is it his proper being!"

It is upon such abstruse speculations as these that Mr. Dendy obviously loves to dwell; he may be said to have a psychological diathesis ever walking, so to speak, within the shadow of his own mind, and pondering upon its mysteries, which, rightly interpreted, suggest to him views in accordance with that pure and holy faith which is its "own exceeding great reward." It is in a blithe and exhilarating tone that Mr. Dendy descants on these subjects; for he has, it would appear, discovered many a verdant spot in what Carlyle calls the "misty sea of metaphysics;" hence the volume before us does not pretend to be a logical disquisition upon the laws of thought—it is written almost in an allegorical spirit, and is replete with poetical associations, fully confirming one of Coleridge's apothegms, that the highest philosophy is identical with the highest poetry. Under these circumstances, criticism is disarmed; we cannot pause to discuss problems which the author only glances at to illustrate opinions which have been entertained by heathen as well as Christian philosophers; we will not dispute with him the spiritualism of Plato, nor the scepticism of Pyrrho, but would rather take refuge in the wit of Sydney Smith, who happily remarks, "Bishop Berkeley destroyed this world in one octavo volume, and nothing remained after his time but mind, which experienced a similar fate from the hand of Mr. Hume, in 1737, so that, with all the tendency to destroy, there remains nothing now left for destruction."* We prefer, as being more consonant with the character of the little volume before us, briefly calling our readers' attention to the way in which our author has treated his subject, which is obviously one of the most difficult he could have selected.

* Elementary Sketches of Moral Philosophy, delivered at the Royal Institution. London: 1850.

The "Birth and Pilgrimage of Thought" is divided into six chapters, and taking Psyche as the root of the Greek word prefixed to each, we have—

| | |
|---|---|
| Psychogenesis . . . | The birth of thought. |
| Psychophrenologia . . | The home of thought. |
| Psychonomia . . . . | The law of thought. |
| Psychopatheia . . . | The bane of thought. |
| Psychotherapeia . . . | The antidote of thought. |
| Psychonoesis . . . . | The force of thought. |

In the first chapter the author briefly refers to the opinions of some of the ancient philosophers concerning the nature of the soul, and repudiates several of the untenable theories of modern sceptics; he then goes back to the history of the creation, and argues that breath or life, soul, and mind, were progressive endowments in the first-born man. This chapter is written in a popular manner, and cannot fail to please the general reader. In the next, the author views his subject under a psychological and physiological aspect, and propounds the following theory:—"We believe," he observes, "that the mind or intellect cannot be an abstraction—cannot be the unity of Brown, or the duality of Wigan and the Alexandrian sophists, or the mere irritability and sensibility of Darwin, which Hunter wisely confined to muscle and nerve, but a *plurality*;" and he adds that he "believes the *plurality* of intellectual organism is proved by the very synchronism of deep and complicated thoughts. If the intellect were an unity, how," he asks, "could Julius Cæsar have compassed the subjects of five letters at once, dictating four to his amanuenses and writing the fifth with his own pen? Or how could Phillidor at once fight and conquer in three chess battles with three antagonists? Or how could Sir Walter Scott dictate a history to young Hogg while his own pen was tracing the labyrinths of a romance?" Mr. Dendy then assumes that "there must not only be a firm and enduring texture of the neurine to *accomplish*, as a rigid muscle will labour without fatigue, but a plurality of organism to arrange and work so long on an idea of thought;" and he adds, that "this plurality of intellect is so clear that the spiritual sophist moves at once our wonder and regret. If mind were a unity, and worked without tissue, the whole series of its manifestations must live and die together; but the persistence of one and the loss or abeyance of another of its faculties, is at once a confutation of the fallacy." (p. 67.) This is ingeniously argued; and as we do not agree with our excellent author, we regret that on this occasion we have not space to discuss the question at issue, which would involve us in a long and serious argument. We have, indeed, cited the passage only with the view of showing Mr. Dendy's opinions on this subject; for it is manifest that, independent of all authorities, he has a disposition to adopt original views, which have, at all events, a strong claim upon our attention. Thus, entertaining the prevailing opinion that the vesicular matter of the cerebral convolutions is associated with the operations of thought, he goes further than any other physiologist, and suggests that "judgment is dependent upon the pure quality of neurine and on the due supply of the pure blood." (p. 81.)

The chapter entitled "Psychopatheia," or the "Light and Shadow of Thought in Emotional Life," refers particularly to the influence of mental emotions upon the physical orgasm, and contains a number of very interesting anecdotes, from which we select the following:

INFLUENCE OF THE MIND ON THE BODY.

"A lady arrived at her home soon after her husband had suddenly died, in consequence of profuse hæmoptysis from a tuberculated lung. An intense and protracted rigor was the first perceptible effect of this shock, and this was followed by a variety of abnormal sensations, especially in the uterine region. I instantly imparted to others my extreme fear that the impulse of thought would be centred on that organ so intimately associated with her deepest sympathies; and the prophecy was true. The child was born a cretin.

"A mother was standing by her child when its clothes caught fire, but was so perfectly paralyzed, that the child was *burned to death*, although there was a tub of water within the mother's reach.

"Two conscript brothers were fighting side by side, when one was killed; the other, on the instant, became an idiot; and the third brother, on the first interview with the idiot, was instantly struck with fatuity, and the two became permanent inmates in the Bicêtre.

"The last sufferer of death for forgery was thus laid prostrate by his sentence, and never rallied. Omichund, on learning the cheat of Lord Clive, became at once an idiot, and died imbecile. A girl was condemned to death by Lord Kenyon, and although the sentence was only *recorded*, she fell lifeless in the dock. Brichteau relates the case of a young officer, who, on the reception of a slight blow, died instantly convulsed: and when Philip V. received the report of the defeat of his army, he sickened and suddenly died.

"A few years ago, just previous to the death of Sir Astley Cooper, he was called in to reconcile the difference of opinion between another surgeon and myself regarding the propriety of operating on the scirrhous breast of a lady, who came from the country, not to *consult* me, but to request me to operate on her at once. Her expressions were most cheerful, and she was evidently buoyed up by a confident hope of speedy relief from the operation. On Sir Astley's announcing, somewhat abruptly, his disapproval of the operation, the lady almost started from her seat, and soon after fainted. From the moment of the return of consciousness, despondency took possession of her thought, and gradually declining, she died in three weeks from the delivery of the verdict."

One of the most interesting chapters is that entitled "Psychonoesis," or "the light and shadow of thought in intellectual life." From this we extract the following passage, which will give our readers a fair idea of the style of our author:

### THE PERILS OF GENIUS.

"The halcyons of intellect may often point to real aberration, if the perils of precocity be not averted. There are many who are marked as the martyrs of thought in youth—like Wm. Pitt and Lord Dudley, who, indeed, 'were never children.' Genius, like beauty, is often a fatal gift: thought not only begins to grow, but bursts into bloom while the organism is as it were still in the bud. By this forcing of the germ, the sensorial ganglion is exhausted of its energy, and the thought that was once a bright and rational thing becomes a chaos or a blank.

"It is these beings who mentally exclaim with Manfred:

——— 'Look on me; there is an order
Of mortals on the earth, who do become
Old in their youth, and die ere middle age.—
Some perishing of study—
And some insanity.'

"The irritability of genius is the first link in that chain of psychical maladies so often terminating in hypochondriasis, when melancholy marks the martyr of thought as its own. The brain of such a being is acutely sensitive, and he shrinks like a mimosa from the breath of criticism. The thoughts of vulgar intellect are a fret to his own, for they have nothing in common. Seneca, we remember, affirms that intellect cannot be happy in society, as the collision would ruffle the courses of its thought. The eye and the mind's eye, the thought, of the astronomer are ever fixed on the 'majestic roof fretted with golden fire;' his thought soars far beyond the influence of the passions and the collision of earth and its people; that earth that, like a Moloch, by a thousand subtle poisons is hourly guilty of infanticide: Halley, and Herschel, and Newton, were octogenarians.

"But the *poetic* thought is almost a creation; and the birth of this thought may often be a convulsive pang of parturition.

"The creation of a *beau-ideal* of thought renders the intellect deeply hypercritical, contented with nothing short of perfection. There was a girl who rejected her real suitors, and died for love of the Belvidere marble. She would sit gazing steadfastly on the Apollo, and strewing flowers over the mosaic steps, and enfolding the statue with a muslin veil of Inde fringed with gold. At length she died raving. The body may become so acutely hyperæsthetic as almost to 'die of a rose in aromatic pain.' The poet's eye that at one time would, like the Titan, scale Olympus, will at another look, like Semele, on Jupiter and all his glory, and perish.

"Of Viotti it is recorded that 'a simple violet would transport him with the liveliest emotion: the slightest impression seemed communicated to all his senses at once, everything spoke to his heart.'

"Those who have contemplated the course of the 'genus irritabile' must feel the deepest sympathy for the penalties of those children of mighty intellect. Ariosto, Dante, Tasso, Alfieri, Voltaire, Rousseau, Cowley, Dryden, Pope, Collins, Johnson, Cowper, Keats, Byron— what a phalanx of beings of bright thought, what a flood of rapture have they rolled into the world of literature, to enlighten and to delight mankind, or to soothe the pillow of anguish. And their own pillow, when the burning brow was laid on that, did the anodyne of slumber always follow? Ask the question of the spirits of those bright meteors that have blazed but to die; the response will be—

'My slumbers, *if* I slumber, are not sleep,
But a continuance of enduring *thought*,
Which then I can resist not: in my heart
There is a vigil, and these eyes but close
To look within.'

"Such a martyr was Paganini. Sleep almost constantly forsook his pillow. His passion almost consumed his being. He felt that his thought was destroying him, but he resigned himself to his fate with the triumphant murmur—'Mais c'est un don du ciel.'"

We regret much that we can only find room for another extract on a subject which cannot be otherwise than deeply interesting to the medical psychologist, who must be well acquainted with the phenomena which are here so well described:

### THE IRRITABILITY OF THOUGHT.

"The irritability of thought is often a consuming fire—a sort of charged jar of intellectual electricity; and the brain finds relief in the safety-valve of exalted composition or acts of absorbing interest. 'The Bride of Abydos' was written by Byron, to keep him from 'going mad, by eating his own heart,' and Reid believed that, if John Howard had not been a philanthropist, he would have been a madman. The eccentric Elia, perchance, had been as mad as his sister, had he not written *hard*. Galileo, close on his 80th year of age, 'could not prevent his restless brain from galloping on.' We may conceive the result, if a curb had been placed on the impetuosity of his thought. Burns was also a martyr to his thought. There were transient gleams of splendour, but his existence was a penalty; it was a sort of champagne vitality. Devoted to the worship of Bacchus or of Venus, he was an enduring slave, either of the Thyrsus or the Cestus. It is perhaps no slight task to decide the struggle between the animal and the intellectual; but in the sensitive, especially, thought *must* gain the victory for intellect, or it will go mad at once—we will not qualify the term. It is true that organism may be so specially *animalized* as to overwhelm a light opposition; but the discipline of thought can effect 'a powerful control, even over those remote organic excitements that so woefully tainted the intellectuality of Burns, who, like the accomplished but wayward Byron, failed in consecrating his licentious pages with his hypothesis of mock morality and virtue.' But, it seems, with all this *esteem* for virtue, Love will be 'Lord of all;' and while it inspired the warm outbreathings of the rough and of the polished child of genius, was uncontrolled by pure thought, and thus the heart of each was reduced to a tainted sepulchre. It is a sad thought that without this erotomania, we should never have revelled in the beautiful episode of the exquisite Haidee, or wept for 'Mary in Heaven.' It is true that the stimulus of Eros may not be in the cerebellum; but there are doubtless two or more conditions somewhere in the organism of those who write morality and practise vice. The most wanton cruelty marked the life of the Dean of St. Patrick's, by which the hearts of three innocent and doting girls were wrung and broken, one dying in her blighted passion; and all the while morality was flowing from his lips and pen. Had this been merely moral insanity, Swift would have been a demon; but we pity as well as condemn, when we know the deep organic disease which was discovered in the brain of Swift.

"We are now emerged from the shadows of the darker ages of the world, when these contrasts and conflicts were referred to the influence of *real* spirits striving for the possession of man's heart. Yet even now the fanatic may affirm that conscience, the good spirit, is whispering virtue in one ear—vice, the evil genius, like Satan, holding up the sensual pleasures of the world as a temptation to crime: while the phrenobiologist will argue that the sound convolution of the brain was the good spirit, the diseased portion the evil genius, and so on.

"Psychology needs not this spurious kind of causation. Her researches discover to her that the excitement, even of a thought, in a soft and sensitive brain, will at once induce various degrees of intellectual disorder, from simple headache to confirmed mania, and this by altering the condition and arrangement of the organism and its circulation."

We are now under the necessity of closing this little volume, which has much interested us, and which we have no hesitation in saying is a very pleasing and graceful contribution to psychological literature.

---

